# Nrf2-Mediated HO-1 Induction Contributes to Antioxidant Capacity of a Schisandrae Fructus Ethanol Extract in C2C12 Myoblasts

**DOI:** 10.3390/nu6125667

**Published:** 2014-12-08

**Authors:** Ji Sook Kang, Min Ho Han, Gi-Young Kim, Cheol Min Kim, Byung Woo Kim, Hye Jin Hwang, Yung Hyun Choi

**Affiliations:** 1 Blue-Bio Industry Regional Innovation Center and Anti-Aging Research Center, Dongeui University, Busan 614-714, Korea; E-Mails: 13839@deu.ac.kr (J.S.K.); bwkim@deu.ac.kr (B.W.K.); hhj2001@deu.ac.kr (H.J.H.); 2 Department of Biochemistry, College of Korean Medicine, Dongeui University, Busan 614-052, Korea; E-Mail: alsgh0615@lycos.co.kr; 3 Laboratory of Immunobiology, Department of Marine Life Sciences, Jeju National University, Jeju 690-756, Korea; E-Mail: immunkim@cheju.ac.kr; 4 Department of Biochemistry, College of Medicine, Busan National University, Yangsan 626-870, Korea; E-Mail: kimcm@pusan.ac.kr; 5 Department of Life Science and Biotechnology, College of Natural Sciences, Dongeui University, Busan 614-714, Korea; 6 Department of Food and Nutrition, College of Human Ecology, Dongeui University, Busan 614-714, Korea

**Keywords:** Schisandrae Fructus, oxidative stress, ROS, Nrf2/HO-1

## Abstract

This study was designed to confirm the protective effect of Schisandrae Fructus, which are the dried fruits of *Schisandra chinensis* (Turcz.) Baill, against oxidative stress-induced cellular damage and to elucidate the underlying mechanisms in C2C12 myoblasts. Preincubating C2C12 cells with a Schisandrae Fructus ethanol extract (SFEE) significantly attenuated hydrogen peroxide (H_2_O_2_)-induced inhibition of growth and induced scavenging activity against intracellular reactive oxygen species (ROS) induced by H_2_O_2_. SFEE also inhibited comet tail formation and phospho-histone γH2A.X expression, suggesting that it prevents H_2_O_2_-induced cellular DNA damage. Furthermore, treating C2C12 cells with SFEE significantly induced heme oxygenase-1 (HO-1) and phosphorylation of nuclear factor-erythroid 2 related factor 2 (Nrf2). However, zinc protoporphyrin IX, a potent inhibitor of HO-1 activity, significantly reversed the protective effects of SFEE against H_2_O_2_-induced growth inhibition and ROS generation in C2C12 cells. Additional experiments revealed that the potential of the SFEE to induce HO-1 expression and protect against H_2_O_2_-mediated cellular damage was abrogated by transient transfection with Nrf2-specific small interfering RNA, suggesting that the SFEE protected C2C12 cells against oxidative stress-induced injury through the Nrf2/HO-1 pathway.

## 1. Introduction

Oxidative stress is a disturbance in the balance between the production of free radicals and antioxidant defense systems. Free radicals are generated in the form of reactive oxygen species (ROS), including superoxide anions, hydrogen peroxide, hydroxyl radicals, and singlet oxygen. Overproduction of ROS accompanied by a reduction in endogenous antioxidative defense causes destructive and irreversible oxidative damage to various cellular components, such as lipids, proteins, and DNA [1,2]. Moreover, increased production of ROS increases oxidative stress, leading to cellular dysfunction and cell death [3,4].

Cells are protected against oxidative stress by either antioxidant enzymes or antioxidant compounds. Among the various antioxidant enzymes, the defense functions of heme oxygenase-1 (HO-1) against oxidative stress have been emphasized [5,6]. HO-1 gene expression is mainly regulated by the nuclear factor-erythroid 2-related factor 2 (Nrf2)-antioxidant response element (ARE) pathway, and induction of this enzyme protects cells against oxidative stress-induced cell death and tissue injury [7,8]. Nrf2 is associated with Kelch-like epichlorohydrin-associated protein 1 (Keap1) under normal conditions and is sequestered in the cytosol. Nrf2 is released from Keap-1 repression under stress conditions and translocates to the nucleus where it transcribes a number of antioxidant and/or detoxification genes, such as HO-1 and NAD(P)H quinone oxidoreductase 1 (NQO-1) [7,9]. Therefore, activation of Nrf2 is critical for cellular rescue pathways against oxidative stress.

Schisandrae Fructus, the dried fruits of *Schisandra chinensis* (Turcz.) Baill. (Schisandraceae), is a popular herbal medicine that has been used extensively in Asia, including Korea, China, Japan, and Russia [10,11]. Schisandrae Fructus is often used to increase physical working capacity and affords stress-protective effects. Schisandrae Fructus and its related compounds possess various biological activities, such as antioxidant, anti-inflammatory, anti-microbial, anti-septic, anti-aging, hepatoprotection, immunostimulating, and anti-cancer effects [12,13,14,15,16]. Although several studies have been conducted on the antioxidant activity of Schisandrae Fructus, the molecular Nrf2/HO-1 signaling pathway by which Schisandrae Fructus exerts antioxidant effects has not been reported. Therefore, we examined the ability of a Schisandrae Fructus ethanol extract (SFEE) to protect C2C12 murine skeletal muscle cells from hydrogen peroxide (H_2_O_2_)-induced cell damage and elucidated the mechanism underlying these protective effects.

## 2. Experimental Section

### 2.1. Preparation of SFEE

Schisandrae Fructus were collected around Mungyeong-city (Gyeongbuk, Korea) and washed three times with tap water before storage at −20 °C. The frozen samples were lyophilized and homogenized using a grinder before extraction. The materials were extracted with 20% ethanol (SFEE) at room temperature for 24 h, filtered, and concentrated using a rotary vacuum evaporator (Buchi Rotavapor R-144, BÜCHI Labortechnik, Flawil, Switzerland). The extract was dissolved in dimethyl sulfoxide (DMSO; Sigma-Aldrich Chemical Co., St. Louis, MO, USA) as a 50 mg/mL stock solution. The stock solution was stored at 4 °C and diluted with medium to the desired concentration prior to use.

### 2.2. Cell Culture and 3-(4,5-dimethylthiazol-2-yl)-2,5-Diphenyltetrazolium Bromide (MTT) Assay

Mouse-derived C2C12 myoblasts were obtained from the American Type Culture Collection (Manassas, VA, USA) and cultured in Dulbecco’s modified Eagle’s medium (Gibco-BRL, Gaithersburg, MD, USA) supplemented with 10% heat-inactivated fetal bovine serum (Gibco-BRL, Gaithersburg, MD, USA), 100 U/mL penicillin G, 100 μg/mL streptomycin, and 0.25 μg/mL amphotericin fungizone at 37 °C in a humid atmosphere of 5% CO_2_ in air. C2C12 cells were assessed by MTT, Sigma-Aldrich Chemical Co., St. Louis, MO, USA) assay as a measure of overall cell viability. C2C12 cells were seeded in 6-well plates at a density of 1 × 10^5^ cells per well. After a 24 h incubation, the cells were treated with various concentrations of the SFEE in the absence or presence of H_2_O_2_ and/or zinc protoporphyrin IX (ZnPP, Sigma-Aldrich Chemical Co., St. Louis, MO, USA) for the indicated times. MTT working solution was added to the culture plates and incubated continuously at 37 °C for 3 h. The culture supernatants were removed completely from the wells, and DMSO was added to dissolve the formazan crystals. Absorbance of each well was measured at 540 nm with a microplate reader (Molecular Devices, Palo Alto, CA, USA). The effect of the SFEE on cell growth was assessed as the percentage of cell viability, where the vehicle-treated cells were considered 100% viable.

### 2.3. Comet Assay (Single-Cell Gel Electrophoresis)

The cell suspension was mixed with 0.5% low melting agarose (LMA) at 37 °C, and the mixture was spread on a fully frosted microscopic slide precoated with 1% normal melting agarose. After the agarose solidified, the slide was covered with 0.5% LMA and immersed in lysis solution (2.5 M NaCl, 100 mM Na-ethylenediaminetetraacetic acid (Na-EDTA), 10 mM Tris, 1% Trion X-100, and 10% DMSO, pH 10) for 1 h at 4 °C. The slides were then placed in a gel electrophoresis apparatus containing 300 mM NaOH and 10 mM Na-EDTA (pH 13) for 40 min to allow for DNA unwinding and expression of alkali-labile damage, and then an electrical field was applied (300 mA, 25 V) for 20 min at 4 °C to draw the negatively charged DNA toward the anode. After electrophoresis, the slides were washed three times for 5 min at 4 °C in a neutralizing buffer (0.4 M Tris, pH 7.5), followed by staining with 20 μg/mL propidium iodide (Sigma-Aldrich Chemical Co., St. Louis, MO, USA). The slides were examined under a fluorescence microscope (Carl Zeiss, Oberkochen, Germany) [17].

### 2.4. Protein Extraction and Western Blot Analysis

After removing the media, the cells were washed with ice-cold PBS and gently lysed for 20 min in ice-cold lysis buffer (40 mM Tris (pH 8.0), 120 mM, NaCl, 0.5% NP-40, 0.1 mM sodium orthovanadate, 2 μg/mL leupeptin, and 100 μg/mL phenymethylsulfonyl fluoride). The supernatants were collected, and protein concentrations were determined using a Bio-Rad protein assay kit (Bio-Rad, Hercules, CA, USA). Equal amounts of the protein extracts were separated on denaturing sodium dodecyl sulphate (SDS)-polyacrylamide gels and transferred electrophoretically to PVDF membranes for Western blotting, (Schleicher & Schuell, Keene, NH, USA). The membranes were incubated overnight at 4 °C with primary antibodies, probed with enzyme-linked secondary antibodies (Amersham, Arlington Heights, IL, USA) for 1 h at room temperature and detected using an enhanced chemiluminescence detection system (Amersham, Arlington Heights, IL, USA). The antibodies were purchased from Santa Cruz Biotechnology (Santa Cruz, CA, USA) and Cell Signaling Technology (Danvers, MA, USA).

### 2.5. Measurement of ROS

The cells were incubated with 10 μM 2′,7′-dichlorofluorescein diacetate (DCF-DA, Molecular Probes, Eugene, OR, USA) for 20 min at room temperature in the dark to monitor ROS production by flow cytometry (Becton Dickinson, San Jose, CA, USA) using Cell-Quest pro software [18].

### 2.6. siRNA Transfection

Nrf2 small interfering RNA (siRNA) and control siRNA were purchased from Santa Cruz Biotechnology (Santa Cruz, CA, USA). The siRNAs were transfected into C2C12 cells according to the manufacturer’s instructions using the Lipofectamine^®^ RNAiMAX transfection reagent (Invitrogen, Carlsbad, CA, USA). The cells were seeded in 6-well culture plates for transfection, and incubated with 50 nM control or Nrf2 siRNA for 6 h in serum-free OPTI-MEM media (Invitrogen, Carlsbad, CA, USA). After the incubation, the transfected cells were subjected to treatment as described in the figure legends.

### 2.7. Statistical Analysis

All measurements were made in triplicate, and all values are presented as mean ± standard deviation. The results were subjected to analysis of variance using Tukey’s test to analyze inter-group differences. In each case, a *p* value < 0.05 was considered statistically significant.

## 3. Results

### 3.1. Effects of the SFEE on C2C12 Cell Viability

**Figure 1:**
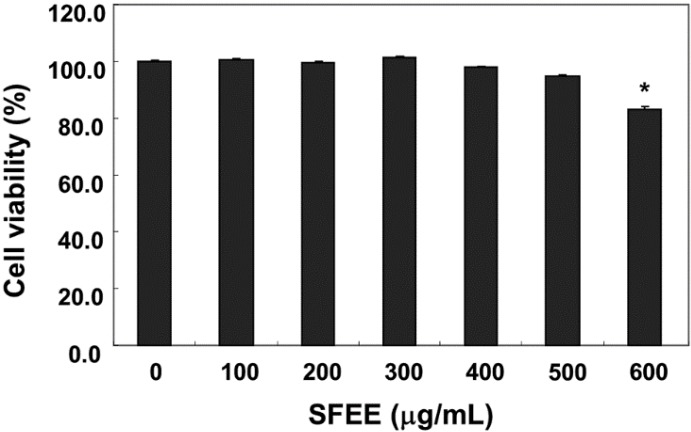
Effects of Schisandrae Fructus ethanol extract (SFEE) on C2C12 cell viability. The cells were treated with various concentrations of SFEE for 24 h. Cell viability was assessed using the MTT reduction assay. Data are mean ± standard deviation of three independent experiments. Differences were determined by Student’s *t-*test (* *p* < 0.05 *vs.* untreated control). SFEE, Schisandrae Fructus ethanol extract; MTT, 3-(4,5-dimethylthiazol-2-yl)-2,5-diphenyltetrazolium bromide.

C2C12 cells were treated with various concentrations of SFEE for 24 h, and cell viability was monitored by the MTT assay to determine the concentration that was not toxic. As shown in Figure 1, 100–500 μg/mL SFEE showed no cytotoxic effects (Figure 1), but the SFEE slightly decreased reduction by MTT at 600 μg/mL. Therefore, 500 μg/mL SFEE was selected as the optimal dose for studying the cytoprotective effect of SFEE against H_2_O_2_-induced cell damage.

### 3.2. SFEE Reduces H_2_O_2_-Mediated Growth Inhibition and DNA Damage in C2C12 Cells

**Figure 2:**
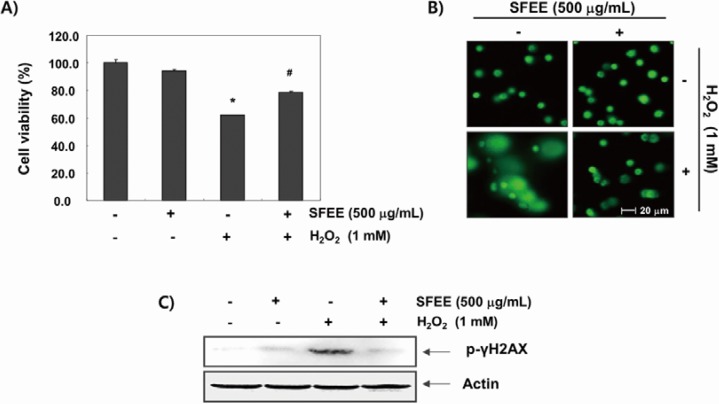
Effects of Schisandrae Fructus ethanol extract on H_2_O_2_-induced growth inhibition and DNA damage in C2C12 cells. (**A**) C2C12 cells were pretreated with 500 μg/mL SFEE for 1 h, incubated with and without 1 mM H_2_O_2_ for 6 h, and cell viability was measured. The results are mean ± standard deviation (SD) values obtained from three independent experiments (* *p* < 0.05 compared with control group; ^#^ *p* < 0.05 compared with H_2_O_2_-treated group); (**B**) The comet assay using cells grown under the same conditions as in (**A**) was performed to detect cellular DNA damage, and representative photographs of the comets were taken using a fluorescence microscope (200 × original magnification); (**C**) The cells were lysed, and equal amounts of the cell lysates were separated on sodium dodecyl sulphate (SDS)-polyacrylamide gels and transferred to nitrocellulose membranes. The membranes were probed with specific antibodies against phospho-histone γH2A.X and actin, as an internal control, and the proteins were visualized using an enhanced chemiluminescent detection system. A representative blot from three independent experiments is shown. SFEE, Schisandrae Fructus ethanol extract.

C2C12 cells were pretreated with 500 μg/mL SFEE for 1 h before being exposed to H_2_O_2_ (1 mM) to investigate the effects of the SFEE against H_2_O_2_-induced oxidative damage. Cell viability was assessed by the MTT assay after 6 h. The results showed that H_2_O_2_ significantly reduced cell viability; however, SFEE significantly and effectively prevented H_2_O_2_-induced cytotoxicity (Figure 2A). Then, we examined H_2_O_2_-mediated damage to C2C12 cell DNA using the alkaline comet assay and Western blotting analyses. Figure 2B shows that a longer comet tail moment (DNA migration) occurred in H_2_O_2_-treated cells, whereas untreated control cells only showed typical representative nuclei. In addition, our results show that H_2_O_2_ alone upregulated the level of phosphorylated histone H2A.X (Ser139) (p-γH2A.X), a classic marker of DNA double-strand break formation [19] (Figure 2C). However, pretreatment with SFEE decreased the number of comet tails and p-γH2A.X expression, indicating a protective effect of SFEE against H_2_O_2_-induced DNA damage.

### 3.3. SFEE Attenuates H_2_O_2_-Induced ROS Generation in C2C12 Cells

**Figure 3:**
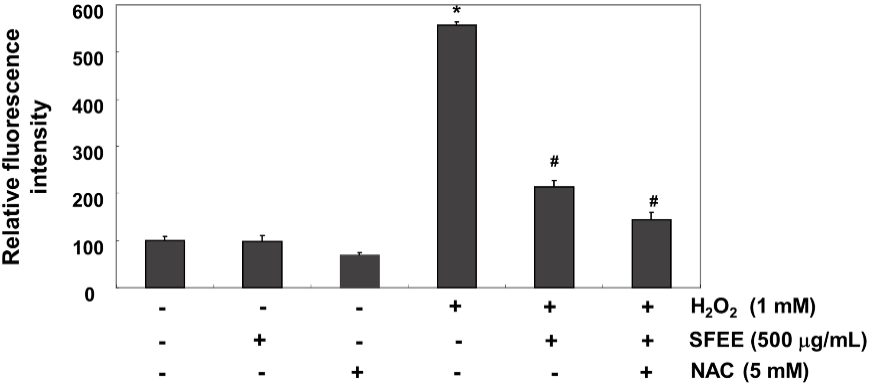
Effects of Schisandrae Fructus ethanol extract on H_2_O_2_-induced reactive oxygen species (ROS) generation in C2C12 cells. C2C12 cells were pretreated with 500 μg/mL SFEE and/or 5 mM N-acetylcysteine (NAC) for 1 h and then stimulated with or without 1 mM H_2_O_2_ for 6 h. The cells were incubated at 37 °C in the dark for 20 min with new culture medium containing 10 μM 2′,7′-dichlorofluorescein diacetate (DCF-DA) to monitor ROS production. ROS generation was measured by flow cytometry. The results are mean ± SD values obtained from three independent experiments (* *p* < 0.05 compared with control group; ^#^ *p* < 0.05 compared with H_2_O_2_-treated group). SFEE, Schisandrae Fructus ethanol extract.

We investigated whether SFEE blocked H_2_O_2_-induced oxidative stress and increased the antioxidative defense system in C2C12 cells using the DCF-DA assay because H_2_O_2_ toxicity was mediated through ROS production. Our results indicated that fluorescence levels of the DCF product, an indication of ROS generation, produced from DCF-DA by ROS increased markedly in H_2_O_2_-treated cells compared with that in untreated cells; however, SFEE significantly reduced H_2_O_2_-induced ROS production (Figure 3). As a positive control, the ROS scavenger *N*-acetyl-l-cysteine (5 mM) also attenuated H_2_O_2_-induced ROS generation, indicating that SFEE scavenged H_2_O_2_-mediated ROS.

### 3.4. SFEE Upregulates Nrf2 and HO-1 Protein Expression

**Figure 4:**
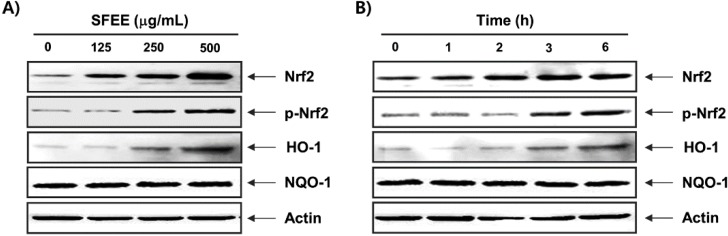
Induction of nuclear factor-erythroid 2 related factor 2 (Nrf2) and heme oxygenase-1 (HO-1) expression by Schisandrae Fructus ethanol extract in C2C12 cells. The cells were incubated with various concentrations of Schisandrae Fructus ethanol extract (SFEE) for 6 h (**A**) or for the indicated periods with 500 μg/mL SFEE (**B**). Cellular proteins were separated on SDS-polyacrylamide gels and transferred to nitrocellulose membranes. The membranes were probed with specific antibodies against Nrf2, p-Nrf2, HO-1, and NAD(P)H quinone oxidoreductase 1 (NQO-1). Actin was used as the loading control.

As Nrf2 is considered a master antioxidant transcription regulator [7,8], we evaluated the regulatory antioxidant potential of SFEE on Nrf2 protein expression by Western blotting. As shown in Figure 4, C2C12 cells exposed to noncytotoxic concentrations of the SFEE caused a concentration and time-dependent increase in Nrf2 expression and phosphorylation levels compared with that in the control group. Furthermore, expression of HO-1, but not NQO-1, which is downstream molecules of Nrf2, also increased following SFEE treatment in a concentration- and time-dependent manner (Figure 4).

### 3.5. SFEE Is Involved with HO-1 in Protecting Against H_2_O_2_

**Figure 5:**
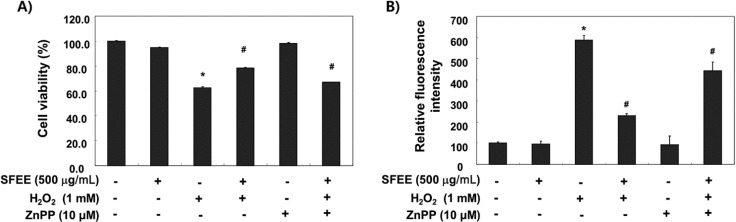
Effects of an HO-1 inhibitor on Schisandrae Fructus ethanol extract-mediated attenuation of growth inhibition and reactive oxygen species (ROS) formation by H_2_O_2_ in C2C12 cells. Cells were pretreated for 1 h with 500 μg/mL SFEE and then treated for 6 h with or without 1 mM H_2_O_2_ in the absence or presence of 10 μM zinc protoporphyrin IX (ZnPP). Then, cell viability (**A**) and ROS generation (**B**) were estimated. Results are mean ± SD values obtained from three independent experiments (* *p* < 0.05 compared with control group; ^#^ *p* < 0.05 compared with H_2_O_2_-treated group). SFEE, Schisandrae Fructus ethanol extract.

An increase in intracellular ROS levels leads to cellular dysfunction and upregulation of HO-1 expression, which plays an important role in protecting against toxicity caused by oxidative insults in a wide variety of cells [5,6]. We blocked HO-1 activity using ZnPP, a selective HO-1 inhibitor, to determine the contributing effect of SFEE by inducing HO-1 expression on its antioxidant capacity. As shown in Figure 5A, the protective effect of SFEE against H_2_O_2_-induced growth inhibition was almost reversed by ZnPP. Furthermore, ZnPP significantly abolished the restoration of ROS generation by SFEE in H_2_O_2_-treated C2C12 cells (Figure 5B).

### 3.6. SFEE Induces Nrf2-Mediated HO-1 Expression in C2C12 Cells

**Figure 6:**
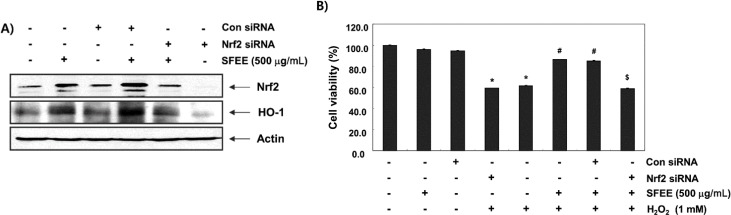
Nuclear factor-erythroid 2 related factor 2 (Nrf2)-mediated induction and heme oxygenase-1 (HO-1) expression by Schisandrae Fructus ethanol extract in C2C12 cells. Cells were transfected with control small interfering RNA (siRNA) and Nrf2 siRNA. After 24 h, the cells were treated with 500 μg/mL Schisandrae Fructus ethanol extract (SFEE) for 6 h (**A**) or pretreated with 500 μg/mL SFEE. for 1 h and then stimulated with or without 1 mM H_2_O_2_ for 6 h (**B**). (**A**) Cellular proteins were separated on SDS-polyacrylamide gels and transferred to nitrocellulose membranes. The membranes were probed with specific antibodies against Nrf2 and HO-1. (**B**) Cell viability was estimated by the 3-(4,5-dimethylthiazol-2-yl)-2,5-diphenyltetrazolium bromide (MTT) assay. Results are mean ± SD values obtained from three independent experiments (* *p* < 0.05 compared with control group; ^#^ *p* < 0.05 compared with H_2_O_2_-treated group; ^$^ *p* < 0.05 compared with H_2_O_2_ and SFEE-treated group).

We knocked down Nrf2 expression using its specific siRNA to further verify the involvement of Nrf2 in SFEE-mediated protection against oxidative stress-induced cytotoxicity. An immunoblot analysis revealed that Nrf2 siRNA reduced basal levels of Nrf2 and HO-1 expression compared with those in untransfected control and control siRNA-transfected cells (Figure 6A). Cells transfected with control siRNA and treated with SFEE showed increased Nrf2 and HO-1 expression levels. However, the increase in the Nrf2 and HO-1 proteins by SFEE was abolished by silencing Nrf2 expression, suggesting that SFEE-mediated upregulation of HO-1 requires activation of Nrf2. Furthermore, Nrf2 siRNA significantly diminished the protective effect of SFEE against the H_2_O_2_-mediated decrease in cell viability (Figure 6B).

## 4. Discussion

Excess ROS generation and/or antioxidant depletion under pathological conditions leads to oxidative stress, along with direct or indirect ROS-mediated DNA adduct, lipid peroxidation, and protein modifications. Although cell and tissue defense systems against ROS consist of various antioxidant enzymes and antioxidants, these defense systems are overpowered in the face of high levels of ROS, which ultimately kill cells via necrosis and/or apoptosis through a variety of overlapping signaling pathways and cascades [7,9]. In this study, we investigated whether SFEE had protective effects against oxidative stress-induced cytotoxicity as part of our screening program for therapeutic antioxidative agents from traditional medicine resources. Our results indicate that SFEE significantly attenuated H_2_O_2_-induced cytotoxicity by decreasing DNA damage in C2C12 cells, supporting that SFEE has antioxidant properties (Figure 2). To further investigate the antioxidative properties of SFEE, we explored the effects of SFEE on H_2_O_2_-induced ROS accumulation and found that SFEE decreased the enhanced ROS generation induced by H_2_O_2_ (Figure 3), indicating that SFEE possesses potent antioxidative stress properties.

The Nrf2/ARE pathway is one of the most important contributors to combat products of oxidation and oxygen radicals [7,8,9]. In response to oxidative stress, Nrf2 is released from Keap1 upon phosphorylation at specific Nrf2 serine and/or threonine residues by several upstream kinases [20,21] and translocated to the nucleus, where it binds to ARE sequences resulting in transcriptional activation of antioxidant genes, such as HO-1 and NQO-1 [7,22]. In particular, there is considerable evidence supporting the role of HO-1, which plays an important role in iron homeostasis as well as antioxidant defense in cells, as a potential target for the control of oxidative stress-induced cellular damage [6,22]. Moreover, induction of HO-1 is recognized as an important therapeutic target for pharmacological intervention of oxidative disorders. Recent reports have demonstrated that phytochemicals can activate Nrf2, resulting in the induction of some cytoprotective proteins including HO-1 [23,24,25,26]. Therefore, we determined the potential role of HO-1 in H_2_O_2_-induced C2C12 cell damage and SFEE-mediated cytoprotection. We have provided evidence for induction of HO-1 by SFEE and showed that SFEE-induced HO-1 protein expression occurred in a concentration- and time-dependent manner, with a concomitant increase in Nrf2 expression as well as its phosphorylation, but not NQO-1 (Figure 4). We further confirmed that induction of HO-1 by SFEE was useful in H_2_O_2_-induced oxidative damage of C2C12 cells using ZnPP, a potent inhibitor of HO-1 activity, and found that ZnPP significantly reduced the protective effect of SFEE against H_2_O_2_-induced ROS generation as well as cytoprotection (Figure 5), thus providing further evidence for HO-1 as a possible cytoprotective pathway for SFEE. These data indicate that activating the Nrf2/HO-1 pathway is closely associated with the SFEE cytoprotective mechanism in C2C12 cells.

Furthermore, the putative roles of the Nrf2/HO-1 pathway on SFEE-mediated cytoprotection against oxidative stress in C2C12 cells were investigated by siRNA-mediated Nrf2 silencing. We found that SFEE-induced Nrf2 and HO-1 expression was effectively reduced by transfection with Nrf2 siRNA. These findings indicate that Nrf2 is an important transcriptional regulator for SFEE-induced HO-1 expression in C2C12 cells. In addition, when Nrf2 was specifically knocked down by siRNA, the restoration of H_2_O_2_-mediated inhibition of C2C12 cell growth was significantly abolished (Figure 6). These results agree with other studies suggesting that Nrf2-dependent HO-1 induction participates in preventing cell oxidative damage and cell survival [23,24,25,26]. Thus, the present data suggest that SFEE upregulates antioxidative enzymes such as HO-1 by activating the Nrf2/ARE pathway, and the SFEE cytoprotective mechanism in C2C12 cells against oxidative stress was critically dependent on activating the Nrf2/HO-1 pathway.

## 5. Conclusions

In summary, our findings demonstrate that SFEE inhibited oxidative stress-induced cellular damage in C2C12 cells. This beneficial effect was closely associated with its potential to eliminate excess ROS accumulation and DNA damage and to activate the Nrf2/HO-1 pathway in H_2_O_2_-treated C2C12 cells. Although our study is limited to emphasize the clinical relevance of SFEE due to the absence of *in vivo* data, upregulation of Nrf2/HO-1 expression may a promising therapeutic target for SFEE during oxidative damage.
